# Efficacy of amitraz acaricide footbaths against cattle and goat tick infestations on sites in Highveld and Lowveld regions of Zimbabwe

**DOI:** 10.1016/j.parepi.2026.e00479

**Published:** 2026-02-03

**Authors:** Obey Daga, Thokozani Hove, Silvester Chikerema, Vladimir Grosbois, Christopher Gadzirai, Frédéric Stachurski, Mathieu Bourgarel, Laure Guerrini

**Affiliations:** a Faculty of Veterinary Science, University of Zimbabwe, Harare, Zimbabwe; b CIRAD, UMR ASTRE, Research Platform Production and Conservation in Partnership, Harare, Zimbabwe; c ASTRE, CIRAD, INRAe, Université Montpellier, Montpellier, France; d Centro de Biotechnologia, Universidade Eduardo Mondlane, Maputo, Mozambique

**Keywords:** Acaricide footbath, Cost-effective tick control, Zimbabwe, Livestock health strategies

## Abstract

Ticks cause significant economic losses in the Zimbabwean livestock sector. Conventional control methods such as plunge dipping or hand spraying are costly, water-intensive, and often impractical, particularly during dry seasons. This study evaluated the efficacy of amitraz acaricide footbaths in reducing tick infestations on cattle and goat across three sites in the Highveld and Lowveld regions of Zimbabwe between February and October 2023. Tick infestation levels were compared between livestock managed using conventional tick control methods (plunge dipping complemented by tick grease application in Lowveld, or complete body hand spraying in the highveld) and livestock managed using footbathing complemented by tick grease application. A total of 21,500 ticks representing eight species were collected on 48 cattle and 48 goats. The effects of treatment and season on tick infestation were tested using zero-inflated negative binomial regression models, which accounted for excess zeros and overdispersion in tick count data. In the Lowveld, footbaths significantly reduced cattle tick infestation by the 3 most abundant tick species: *Ripicephalus microplus* (40% reduction), *Amblyoma. hebraeum* (51%) and *R. decoloratus* (43%). Significant reductions in infestation rate by *A. hebraeum* (70%) and *R. decoloratus* (68%) was also observed on goats from the same Lowveld site. In the Highveld goat site, footbath significantly reduced infestation by *A. hebraeum* (91%), *R. appendiculatus* (46%), *R. decoloratus* (49%) and *R. evertsi evertsi* (82%) on goats. In the Highveld cattle site where the conventional method to control ticks was complete body hand spraying, footbathing was not more effective than the conventional method for controlling *R. decoloratus*, the dominant tick species. Tick counts varied seasonally, with *Rhipicephalus* subgenus *Boophilus* ticks most abundant on cattle, during the dry season in Lowveld and during the rainy season in Highveld). Similarly, footbath did not perform better than conventional methods to control Hyalomma tick species (i.e. *H. truncatum* and *H. rufides*) found in goats and cattle in the Highveld and Lowveld sites. The study also shows patterns of seasonal variation (i.e. difference between the rainy season and the dry season) in ticks infestation rates that differ depending on site, host species and tick species. These findings suggest that acaricide footbaths provide a practical, low-cost alternative for tick control in both cattle and goats, especially in areas with limited water resources.

## 1 Introduction

Ticks are hematophagous ectoparasites that can transmit disease-causing pathogens during their feeding process (Jain et al., 2021). Eighty per cent of the world's livestock is at risk of infection by ticks and tick-borne diseases (TBDs) particularly in tropical and subtropical regions (Ahmad et al., 2025; Amin and Sulabh, 2025; De León et al., 2021; Rahali et al., 2025; Yawa et al., 2020). In Zimbabwe, TBDs account for more than 60% of disease-related cattle mortality(Mavedzenge et al., 2006; Sungirai et al., 2015; Tavirimirwa et al., 2013; Yawa et al., 2020) posing significant economic and health challenges for the livestock sector. Beyond pathogen transmission, ticks cause direct harm through skin wounds, secondary bacterial infections, on attachment sites, exsanguination leading to low productivity and production of toxins (Jongejan and Uilenberg, 2004; Moyo, 2015; Sungirai et al., 2015). In dairy animals, tick bites can lead to irreversible udder damage (Ndhlovu et al., 2009; Ndlovu, 2014; Walker, 2003; Young et al., 1988). Effective and sustainable tick control strategies are therefore essential.

In Zimbabwe, tick control primarily relies on topical acaricide application via plunge dipping or hand spraying(DVS, 2022). Other methods, such as pour-on acaricides or injectable macrocyclic lactones, are used infrequently and mostly by resourced or large scale farmers. Most livestock owners rely on plunge-dipping vats that date back to the early 20th century and now require significant renovations (Masuku et al., 2015). These vats consume large volumes of water (∼15,000 L per session) and acaricide (∼ 1500 g/dipping session), making them costly and environmentally damaging. In marginalised areas, plunge dipping becomes impractical due to water scarcity, particularly in the dry season. Additionally, under-dosing due to high acaricides costs fosters the selection of acaricide-resistant tick populations (Githaka et al., 2022; Sichibalo et al., 2021). Following Zimbabwe's land reform program, many small and medium-sized farms lack nearby dipping infrastructure, leaving livestock inadequately protected. These challenges underscore the need for innovative, cost-effective, and environmentally sustainable tick control methods adaptable to various farming contexts (Walker, 2003; Young et al., 1988).

In Southern Africa, cattle and goats are major livestock of economic and cultural importance (Jongejan et al., 2020; Mapiye et al., 2019) and livestock rearing is a prime source of rural communities and low-income groups livelihoods (Jain et al., 2020a). In Zimbabwe, whilst cattle tick control is a major legislated animal health activity, limited effort is directed at goats reared in marginalised communal farms, which account for over 97% of the country's total goat population (Christopher et al., 2020). Goats have a significant role in rural household food security, liquid capital, and cultural ceremonies. Goats can adapt to the harsh environments that are being ushered by climate change and could be the best livestock option in disaster rebound (Akinmoladun et al., 2019; Mkwanazi et al., 2020). In Zimbabwe, at least 14 tick species were found infesting goats (Hove et al., 2008; Assan and Sibanda, 2014) and 13 species for cattle (Sungirai et al., 2015). Goats can succumb to TBDs such as heartwater, caused by *Ehrlichia ruminantium,* which can be transmitted by vectors to other ruminant species. Conventional tick control measures, such as plunge dipping, are generally not implemented for goats in the same manner as in cattle (Spickett and Fivaz, 1992; Nyangiwe and Horak, 2007). Goats, typically not dipped, may act as reservoirs for some ticks and tick-borne pathogens that parasitise other ruminants (Nyangiwe and Horak, 2007; Jongejan et al., 2020). A tick control strategy adapted to large but also small ruminants, such as goats or sheep, is thus required.

Ticks such as *Hyalomma* spp. and *Amblyomma* spp. prefer attaching to the legs, feet, perianal, and ventral regions of their hosts (Kok and Fourie, 1995; Hove et al., 2008; Selmi et al., 2025; Tharwat et al., 2024; Walker, 2003), often causing lameness and physiological alterations in small ruminants. These species are prolific and can cause substantial hide damage due to their clustering and deep-feeding mouthparts. Notably, *Amblyomma variegatum* initially attaches to inter-digital spaces before migrating to preferred feeding sites during rest (Stachurski, 2000). This early-stage behaviour offers a window for targeted treatment. Therefore, exploiting this attachment behaviour forms the biological rationale for testing the efficacy of acaricide footbaths in reducing tick infestations. Acaricide footbaths apply treatment directly to the animal's feet, an approach shown to reduce cattle *A. variegatum* infestations in West Africa (Stachurski and Lancelot, 2006). Their use in tick control in sheep and goats has also been reported in South Africa (Spickett and Fivaz, 1992). Footbaths offer several advantages: they conserve water (300 L) and acaricide, are low-cost, easy to construct and maintain, and suitable for use in dry seasons and marginal areas.

This study aimed to evaluate the efficacy of acaricide footbaths in reducing tick infestations on cattle and goats in Zimbabwe. Specifically, we measured (i) the reduction in infestation intensity of *A. hebraeum*, the predominant bont tick, and (ii) the effect on other prevalent tick species.

## 2 Methodology

### 2.1 Study sites

**Fig. 1:**
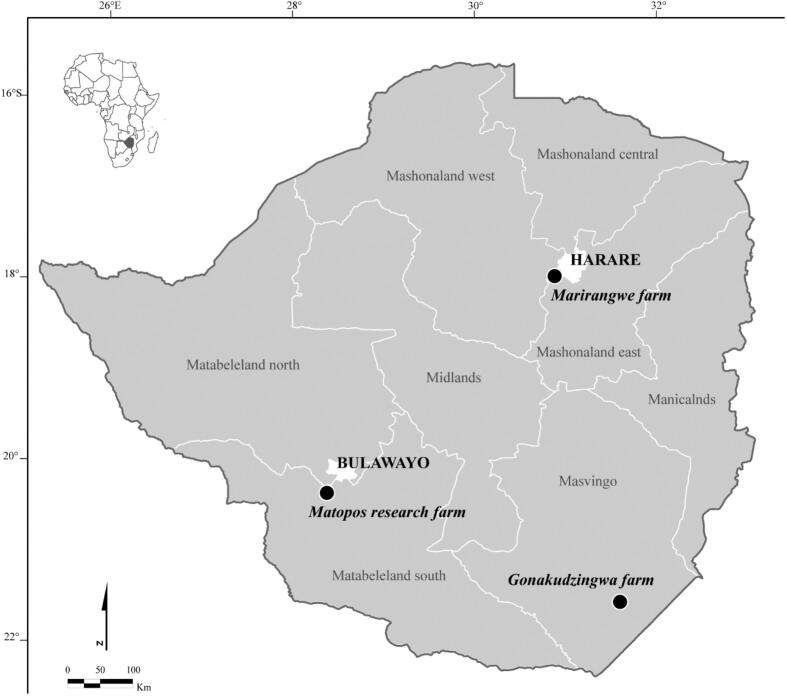
Location of study sites in Zimbabwe.

The study was conducted in the Highveld and Lowveld areas of Zimbabwe from February to October 2023. The study sites were selected considering primarily logistical feasibility and convenience (mainly farmer willingness to participate and accessibility). However, these study sites were characterized by contrasted livestock production systems and ecological characteristics (see below) which allowed the assessment of footbath effectiveness not only in different contexts. Two Highveld sites were chosen to represent different livestock production types, while one Lowveld site allowed assessment under contrasting climatic and vegetation conditions. The locations of the study sites are presented in Fig. 1.

#### 2.1.1 Highveld - Marirangwe

The highveld footbath trial was conducted at Marirangwe farm, located in Beatrice (approximately 45 km southwest of Harare) at an altitude of 1450 m. The site lies in a high-potential farming area characterized by *Terminalia* wooded grassland with predominantly sour veld, where *Sporobolus pyramidalis* dominates near pens and paddocks. Annual rainfall ranges from 750 to 1000 mm (November–April), and mean temperatures are 16–19 °C. The farmer engages in mixed beef and dairy cattle farming, and only cattle were sampled in this study.

#### 2.1.2 Highveld - Matopos research, Longsdale farm

The highveld goats' footbath trial was conducted at Matopos Research Institute, located at an altitude of 1340 m, approximately 30 km south of Bulawayo, in an area of high livestock production potential. Vegetation consists of *Acacia* woodland with sour veld dominated by with *Hyparrhenia, Heteropogon, Brachiaria, Panicum*, and *Eurocloa* grasses. Average annual rainfall is 580 mm, and mean temperatures range from 18 °C to 24 °C.

#### 2.1.3 Lowveld - Gonakudzingwa farm

The lowveld cattle and goats' footbath trial was conducted at Gonakudzingwa Farm, located in Chiredzi district along the peripheries of Gonarezhou National Park, approximately 90 km west of Chiredzi town. The site lies at 480 m altitude in a high livestock production potential area,. Average annual rainfall is about 500 mm (November – April) and mean annual temperatures range from 21 to 25 °C. Vegetation consists of mixed *Mopane-Combretum-Dychrostachys* woodland with *Grewia* shrubs and grasses dominated by *Eurocloa, Brachyaria, Panicum, Digitaria*, *Aristida, Eragrostis* and *Setaria* species, indicating a mixed sweet-sour veld. The proximity of this site to national parks gives the tick vectors abundant alternative wild hosts for their perpetuity, making livestock tick control more complex.

### 2.2 Sampling per site

At each site, a total of 24 animals per species were selected, with 24 cattle at Marirangwe farm, 24 goats at Matopos and 24 each for cattle and goats at Gonakudzingwa farm. Animals were stratified and randomly allocated into two groups of 12 to form the treatment and control groups. Each animal was ear-tagged with a unique identity number. Cattle ranged from 2 to 10 years, while goats included all age categories.

### 2.3 Animal management

• For each farm and species, animals were randomly allocated to either the footbath treatment group or a control group. Both groups were kept under the same environmental and grazing conditions, sharing naturally tick-infested rangeland. Due to logistical constraints and limited penning facilities at the study sites, treatment and control animals were sometimes housed together overnight. This setting was unavoidable; however, animals were randomly allocated to either group and individually so that it was not possible for an animal belonging to one group to be subjected to the treatment method of the other group. Footbath animals walked through an acaricide footbath in the evenings after grazing, every second day during the rainy season and every third day during the dry season. Control animals received standard community-based tick control measures (plunge dipping or hand spraying), in line with common practices for the rest of the herd. Because housing both groups together could potentially allow limited tick transfer potentially resulting in homogenization of infestation levels among the two treatment groups, the detection of a significant difference in tick counts significant reductions in tick counts were consistently observed on foot-bathed animals.

### 2.4 Acaricides used

Amitraz wettable powder (Amitik, Coopers Zimbabwe and Agroshape Zimbabwe) was used for the footbath treatments. Weekly total replacement (TR) was applied for chemical replenishment and lime, which usually comes as a combo with the amitik chemical, was used for PH maintenance of the bath. Based on the Department of Veterinary Services' recommendations, drawn from experience with amitraz application and considering supplier market proximity, emulsified acaricide concentrations of 50% and 25% were used in the Lowveld and Highveld regions, respectively. Conventional tick control methods varied by region and species: hand spraying was practiced on cattle at Highveld Marirangwe site (weekly during the dry season and on 5.5.4 days schedule in the rainy season); plunge dipping was employed on cattle (weekly and fortnightly during the rainy and the dry season, respectively) in the Lowveld Gonakudzingwa site; no acaricide was applied on goats in the Lowveld Gonakudzingwa site; plunge dipping was employed fortnightly, only during the rainy season, on goats from the Matopos Research site. Additionally, tick grease containing deltamethrin 0.1%m/m and piperonyl butoxide 0.5%m/m (Coopers Zimbabwe), provided through a government supply program, was applied to all cattle in both treatment and control groups at predilection sites (ears and horn bases) for *Rhipicephalus appendiculatus*. This measure was applied uniformly to all animals and served as a complementary intervention to reinforce conventional tick control strategies to limit *R. appendiculatus* infestation and reduce the risk of theileriosis transmission. Tick grease application was implemented primarily to protect farmers' cattle during a peak theileriosis outbreak in Zimbabwe, and previous studies have shown that this method alone does not effectively control *R. appendiculatus*, which transmits the disease.

### 2.5 Animal handling for tick collection

To facilitate tick collection and counting, Marirangwe cattle were handled in a race, and Gonakudzingwa cattle were either handled in a race or by rope restraint (halters). Goats at both Gonakudzingwa and Matopos were hand-restrained.

### 2.6 Tick collection and counting

Tick collections and counts were done during the rainy season (February to April 2023) and dry season (July to October 2023). Collections were performed in the morning, a day before the farm's scheduled conventional dipping day, weekly for cattle and fortnightly for goats. Adult and nymph ticks were collected from the whole body of both cattle and goats using steel forceps and transferred to plastic vials containing a 70% ethanol solution as preservation media. Each tube was labelled with the study site, collection date, and animal identification number. Samples were transported to the University of Zimbabwe, Faculty of Veterinary Science Parasitology laboratory for species identification and count.

### 2.7 Tick identification

Ticks collected from cattle and goats were morphologically identified to species level using standard taxonomic keys and a stereomicroscope (Walker, 2003; Horak et al., 2018). Identification was performed at the Zimbabwe National Central Veterinary Laboratory (CVL), which specializes in tick taxonomy. To ensure accuracy, the same samples were validated by a professor at the University of Zimbabwe Veterinary Parasitology Laboratory.

### 2.8 Statistical analysis

The objectives of the statistical analysis were to depict for each tick species, host species and study site, the variation in tick infestation according to treatment and season, to provide corresponding estimations of tick infestation and to test the difference in infestation between animals submitted to the footbath and the conventional control treatments.

As a first step, the best fitting distribution for tick counts was identified for each study site, tick species, and host species by fitting Poisson, zero-inflated Poisson, and zero-inflated negative binomial distributions to the observed count frequency data. Zero-inflated models were chosen when data exhibited overdispersion and excess zeros, which standard Poisson or negative binomial models could not adequately account for. The Akaike Information Criterion (AIC) was used to evaluate and compare the adequacy of these distributions. In the second step, for each combination of tick species, site, and host species with a sufficient number of observed ticks, a statistical model was developed. This model assumed the distribution selected in the first step for the count response variable and included the effects of season, treatment, and their interaction. The model was performed using the Political Science Computational Laboratory (pscl) R package in R version 4.3.1. Finally, an iterative descending model selection procedure was applied. At each iteration, a Likelihood Ratio Test (LRT) was used to determine the statistical significance of the terms in the model. The term with the largest non-significant *P*-value was removed. During this procedure, the main effect terms were tested only when the interaction had already been removed. The procedure ended when the LRT P-value associated with each remaining term in the model was ≤0.05.

## 3 Results

A total of 21,500 ticks, encompassing all species collected from both cattle and goats, were recorded throughout the study. Of these, 1152 ticks were collected during the initial pre-treatment assessment (Table 1). At this baseline stage, infestation levels were similar between animals in the two study groups: the control group, treated using conventional plunge dipping, hand spraying, or not using any acaricide treatment depending on the site and species, and the treatment group, subjected to the acaricide footbath (all treatment group effect *p*-values>0.05; Table 1). This indicates that there were no significant differences in tick burden prior to the application of treatments.

**Table 1:** Baseline tick counts per tick species, host species, and sites. When data allowed (only for cattle and some but not all tick species), Likelihood Ration Test *p*-value for difference in tick count between the animals from the conventional and the footbath groups before the onset of the experiment was determined by fitting a zero-inflated negative binomial model including only a treatment effect on the count component and a zero-inflated negative binomial model including only intercepts.

		Lowveld- Gonakudzingwa					Highveld- Marirangwe			Highveld- Matopos	
		Cattle			Goat		Cattle			Goat	
	Treatment	C	FB	Treatment P-value	C	FB	C	FB	Treatment *P*-value	C	FB
**Tick species**	**Ah**	319	321	0.78	2	3	0	0	–	1	2
	**Ht**	64	52	0.61	0	0	3	11	0.06	0	0
	**Hr**	30	29	0.90	0	0	0	0	–	0	0
	**Rd**	18	13	0.72	0	2	18	30	0.40	0	0
	**Rm**	0	0	–	0	0	0	0	–	0	0
	**Re**	0	0	–	0	1	0	0	–	0	2
	**Rz**	0	0	–	0	0	0	0	–	0	0
	**Ra**	107	119	0.88	1	0	0	0	–	3	1

Ah: *A. hebraeum;* Ht: *H. truncatum* Hr*: H. rufipes,* Rd*: R.decoloratus,* Rm*: R.microplus,* Re*: R. evertsi evertsi,* Rz*: R. zambeziensis,* Ra*: R. appendiculatus.*

### 3.1 Lowveld Gonakudzingwa – Cattle and Goats

A total of 11,143 ticks (Table 1, Table 2) were recorded mainly on cattle (10657) but also on goats (486). Eight tick species were represented: *Rhipicephalus. microplus* (3725 ticks representing 33% of collected ticks), *Amblyoma hebraeum* (3607 ticks: 32% of collected ticks), *Rhipicephalus decoloratus* (1864 ticks: 18% of collected ticks), *Hyalomma truncatum* (1108 ticks: 10% of collected ticks)*, Rhipicephalus appendiculatus* (593 ticks: 4% of collected ticks), *Hyalomma rufipes* (129 ticks: 1% of collected ticks)*, Rhipicephalus zambeziensis* (94 ticks: <1% of collected ticks) and *Rhipicephalus evertsi evertsi* (23 ticks: <1% of collected ticks).

**Table 2:** Tick counts following the start of the experiment per tick species, host species, site and season,

Site	Lowveld-Gonakudzingwa										Highveld-Marirangwe					Highveld-Matopos				
Host type	Cattle					Goat					Cattle					Goat				
Season	Rainy (12*)		Dry (14*)		Δ%^&^	Rainy (11*)		Dry (8*)		Δ%^&^	Rainy (14*)		Dry (15*)		Δ%^&^	Rainy (4*)		Dry (8*)		Δ%^&^
Treatment	C	FB	C	FB	C-FB	C	FB	C	FB	C-FB	C	FB	C	FB	C-FB	C	FB	C	FB	C-FB
Ah	772	474	1019	406	51	68	15	155	53	70	1	0	1	0		17	1	51	5	91
Rd	484	190	659	461	43	14	3	14	6	68	3080	4299	1271	1395	-31	10	15	39	10	49
Rm	323	123	1970	1245	40	26	7	15	16	44	0	0	0	0		0	0	0	0	
Ra	166	94	31	49	27	6	3	12	5	56	0	0	4	14	−250	6	6	35	16	46
Rz	13	47	15	15	−120	1	0	3	0		0	0	0	0		0	0	0	0	
Re	2	3	9	6	18	0	1	0	1		0	0	0	0		2	2	31	4	82
Ht	374	377	123	66	11	17	32	2	1	−74	24	75	30	24	−83	3	0	2	0	
Hr	22	26	13	8	3	1	0	0	0		3	3	8	3	45	0	0	0	0	

NB. The majority of these ticks (>95%) were adults (data not shown) except for nymphs of the *Amblyomma* species which were collected from goats.

Ah: *A. hebraeum;* Rd*: R.decoloratus,* Rm*: R.microplus, C,* Ra*: R. appendiculatus,* Rz*: R. zambeziensis*, Re*: R. evertsi evertsi,* Ht: *H. truncatum* Hr*: H. rufipes*. Treatment: C: Conventional; FB: Footbath; * number of weekly sampling sessions per season, ^**&**^ Difference between number of ticks collected across seasons under conventional treatment and number of ticks collected across seasons under footbath treatment expressed as fraction in percent of number of ticks collected across seasons under conventional treatment.

#### 3.1.1 Cattle

**Fig. 2:**
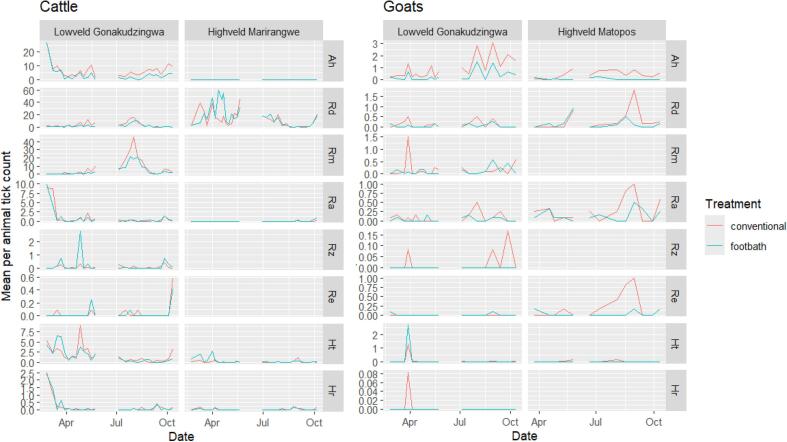
Mean number of collected ticks in cattle and goats across the study period.

**Fig. 3:**
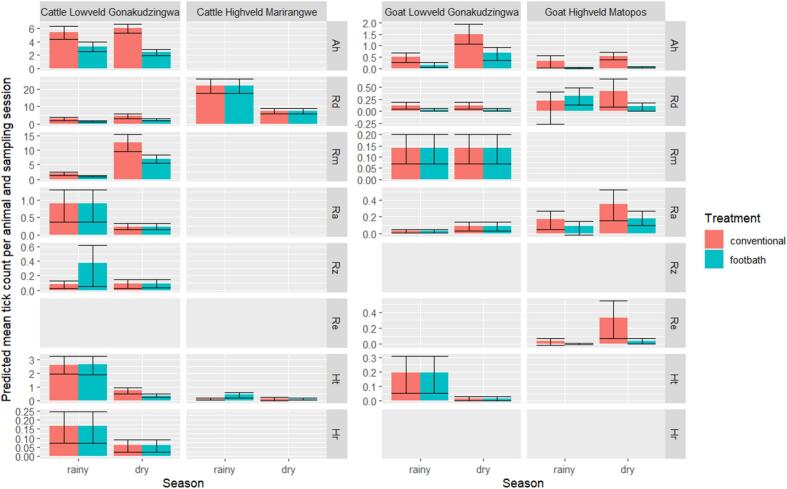
Predicted mean tick counts and 95% confidence intervals for the most abundant tick species at each study site.

Tick counts on cattle varied significantly between the rainy and the dry seasons for most tick species (Table 3) with different patterns according to the tick species (Fig. 2, Fig. 3): tick counts were higher during the rainy season than during the dry season for *H. truncatum* (zero-inflation component *p* < 0.001, treatment by season count component interaction *p* = 0.024, Table 3, Fig. 3), *R. appendiculatus* (count component *p* < 0.00001, Table 2, Table 3, Fig. 2, Fig. 3), *H. rufipes* (count component *p* < 0.01, Table 2, Table 3, Fig. 2, Fig. 3) and *R. zambeziensis* (treatment by season count component interaction *p* = 0.03, Table 2, Table 3, Fig. 2, Fig. 3) while it was higher during the dry season than during the rainy season for *R. decoloratus* (count component p < 0.01, Table 2, Table 3, Fig. 2, Fig. 3) and *R. microplus* (count component p < 0.00001, Table 2, Table 3, Fig. 2, Fig. 3)*.* Seasonal pattern was less clear for *A. hebraeum*: despite a significant effect of season on the zero-inflation component (*p* = 0.03, Table 3) and a significant treatment by season interaction on the count component (*p* < 0.001, Table 3), overall tick count did not differ noticeably between the rainy and the dry season (Fig. 2, Fig. 3)*.*

**Table 3:** Likelihood ratio test p-values during the selection of zero-inflated negative binomial models fitted to tick counts by host species, study sites and tick species.

Model component	Count component			Zero inflation component		
	Treatment	Season	T:S Interaction	Treatment	Season	T:S Interaction
**Lowveld-Cat**						
Ah	_	_	**<0.001**	**<0.001**	**0.03**	0.67
Rd	**<0.001**	**<0.01**	0.06	0.63	0.97	0.98
Rm	**<0.00001**	**<0.00001**	0.07	0.55	**<0.00001**	0.94
Ra	0.79	**<0.00001**	0.07	0.96	0.96	0.95
Rz	_	_	**0.03**	0.85	0.32	0.32
Ht	_	_	**0.024**	0.71	**<0.001**	0.68
Hr	0.78	**<0.01**	0.39	0.96	0.98	0.97
**Lowveld-goats**						
Ah	0.06	**0.023**	0.82	**<0.001**	**<0.01**	0.23
Rd	**0.02**	0.20	0.47	0.64	0.57	0.60
Rm	0.20	0.58	0.13	0.97	0.97	0.97
Ra	0.09	**0.02**	0.97	0.4	0.96	0.98
Ht	0.34	**<0.001**	0.39	0.99	0.94	0.97
**Highveld-Cattle**						
Rd	0.11	**<0.00001**	0.36	0.88	0.58	0.77
Ht	_	_	**<0.01**	0.99	0.96	0.96
**Matopos-Goats**						
Ah	**<0.00001**	**0.03**	0.67	0.95	**<0.01**	0.97
Rd	_	_	**0.032**	0.98	0.99	0.98
Ra	**0.05**	**0.05**	0.21	0.65	0.96	0.75
Re	**<0.001**	**<0.01**	0.99	0.98	0.99	0.99

Whenever the treatment by season interaction was statistically significant (i.e. p-value<0.05), the p-values of the main effects (Treatment and Season) were meaningless and were therefore omitted (marked as “–”).

Tick species: Ah: *A. hebraeum;* Ht: *H. truncatum* Hr*: H. rufipes,* Rd*: R.decoloratus,* Rm*: R.microplus, C,* Re*: R. evertsi evertsi,* Rz*: R. zambeziensis,* Ra*: R. appendiculatus*.

Tick counts were significantly lower for cattle treated with the acaricide footbath than for cattle receiving the conventional plunge dipping treatment for *A. hebraeum* (880 ticks compared to 1791 ticks: 51% difference, Table 2, zero-inflation component p < 0.001, count component treatment by season interaction p < 0.001, Table 3)*, R. decoloratus* (651 ticks compared to 1143: 43% difference, Table 2, count component *p* < 0.001, Table 3), *R. microplus* (1368 ticks compared to 2293: 40% difference, Table 2, count component p < 0.00001, Table 3) and to a lesser extent *H. truncatum* (443 ticks compared to 497: 11% difference, Table 2, treatment by season count component interaction p < 0.00001, Table 3). The significant treatment by season interaction on the count component for *A. hebraeum* and *H. truncatum* reflected a larger difference in tick counts between the footbath and conventional treatment groups during the dry season than during the rainy season (Fig. 3).

No statistically significant differences in tick counts between the footbath and the control group were observed for *R. appendiculatus* (zero-inflation component *p* = 0.96, count component *p* = 0.79, Table 3) and *H. rufipes* (zero-inflation component p = 0.96, count component *p* = 0.78, Table 3).

Ticks count were significantly higher for cattle treated with the acaricide footbath than for cattle receiving the conventional plunge dipping treatment for *R. zambeziensis* (62 ticks compared to 28 ticks: 120% difference, Table 2, count component treatment by season interaction p = 0.03, Table 3). The treatment by season interaction for *R. zambeziensis* reflected higher tick counts for the footbath group than for the conventional group during the rainy season only, while no difference was observed during the dry season (Table 2 Fig. 2, Fig. 3).

#### 3.1.2 Goats

Tick counts on goats in the Gonakudzingwa site varied significantly between the rainy season and the dry season for *A. hebraeum* (zero-inflation component *p* < 0.01, count component *p* = 0.023, Table 3), *H. truncatum* (count component p < 0.001, Table 3) and *R. appendiculatus* (count component p = 0.02, Table 3). Seasonal pattern differed among tick species with higher, higher tick counts on goats during the dry season for *R. appendiculatus* and *A. hebraeum* while higher tick counts were observed during the rainy season for *H. truncatum* (Fig. 2, Fig. 3). No significant seasonal variation in tick counts on goats in Gonakudzingwa was detected for *R. microplus* and *R. decoloratus* (Table 2, Table 3, Fig. 2, Fig. 3).

Tick counts were significantly lower for goats treated with the acaricide footbath than for goats treated under the conventional regime (i.e. no acaricide treatment) for *A. hebraeum* (68 ticks compared to 223, 70% difference, Table 2, zero-inflation component p < 0.001, count component *p* = 0.06, Table 3, Fig. 2, Fig. 3) and *R. decoloratus* (9 ticks compared to 28: 70% difference, Table 2, count component p = 0.02, Table 3, Fig. 2, Fig. 3).

The number of ticks collected was not significantly lower for goats treated using the footbath than for goats under the conventional regime (i.e. no acaricide treatment) for *R. microplus* (zero-inflation component *p* = 0.97, count component *p* = 0.20, Table 2, Table 3, Fig. 2, Fig. 3), *R. appendiculatus* (zero-inflation component *p* = 0.4, count component *p* = 0.09, Table 2, Table 3, Fig. 2, Fig. 3) and *H. truncatum* (zero-inflation component *p* = 0.99, count component *p* = 0.34, Table 2, Table 3, Fig. 2, Fig. 3).

*3.2 Highveld, Marirangwe - Cattle.*

10,297 ticks were collected on cattle exclusively at the Marirangwe site (Table 1, Table 2). Most of them were *R. decoloratus* (10,093 ticks representing 98% of collected ticks), followed by *H. truncatum* (167 ticks: 1.5% of collected ticks), *R. appendiculatus* (18 ticks: <1% of collected ticks), *H. rufipes* (17 ticks: <1% of collected ticks), and *A. hebraeum* (2 ticks).

While *R. decoloratus* ticks in the Marirangwe site were clearly and significantly (*p*-value <0.00001 for the count component Table 3) more abundant during the rainy season than during the dry season (Table 2, Fig. 2, Fig. 3), the seasonal pattern was less clear for *H. truncatum*: a significant treatment by season interaction on the count component (*p* < 0.01, Table 3) reflected contrasted pattern between the footbath treatment group (higher tick counts during the rainy season Fig. 2, Fig. 3) and the conventional treatment group (no noticeable difference between the rainy and the dry seasons Fig. 2, Fig. 3)*.*

The footbath treatment did not perform better than the conventional hand spraying method for controlling infestation by *R. decoloratus* on cattle in the Marirangwe site (*p*-values = 0.11 and 0.88 for the count and the zero-inflation component, respectively Table 3). The same conclusion was reached on that site for cattle infestation by *H. truncatum* with the detection of a significant count component season by treatment interaction (p < 0.01, Table 3): ticks counts were higher for the footbath treatment group during the rainy season while no difference was detected during the dry season (Fig. 2, Fig. 3).

### 3.2 Highveld, Matopos - Goats

264 ticks were collected on goats exclusively at the Matopos site (Table 1, Table 2). Five tick species were identified: *A. hebraeum* (77 ticks representing 29% of collected ticks), *R. decoloratus* (74 ticks: 28% of collected ticks), *R. appendiculatus* (67 ticks: 25% of collected ticks), *R. evertsi evertsi* (41 ticks: 16% of collected ticks) and *H. truncatum* (5 ticks: 2% of collected ticks).

Tick counts on goats in the Matopos site varied significantly between the rainy season and the dry season for *A. hebraeum* (zero-inflation component p < 0.01, count component *p* = 0.03, Table 3), *R. appendiculatus* (count component *p* = 0.05, Table 3) and *R. evertsi evertsi* (count component p < 0.01, Table 3). For these three species ticks were more abundant during the dry season than during the rainy season (Fig. 2, Fig. 3). The seasonal pattern for tick count on goats in Matopos was less clear for *R. decoloratus*: a significant count component treatment by season interaction (*p* = 0.032, Table 3) reflected differing seasonal patterns between the footbath treatment group (higher tick counts during the rainy season, Fig. 2, Fig. 3) and the conventional treatment group (higher tick counts during the dry season, Fig. 2, Fig. 3).

In Matopos, tick counts were significantly lower for goats treated with the acaricide footbath than for cattle receiving the conventional plunge dipping treatment for *A. hebraeum* (6 ticks compared to 68 ticks: 91% difference, Table 2, count component *p* < 0.00001, Table 3)*, R. decoloratus* (651 ticks compared to 1143: 43% difference, Table 2, count component *p* < 0.001, Table 3), *R. appendiculatus* (41 ticks compared to 22: 46% difference, Table 2, count component p = 0.05, Table 3), *R. evertsi evertsi* (33 ticks compared to 7: 82% difference, Table 2, count component p < 0.001, Table 3) and *R. decoloratus* (49 ticks compared to 25: 49% difference, Table 2, count component treatment by season interaction p = 0.032, Table 3). The significant treatment by season interaction on the count component for *R. decoloratus* lower tick counts for the footbath treatment group than for the conventional treatment group during the dry season only (Fig. 2, Fig. 3).

## 4 Discussion

The present study demonstrates higher effectiveness of footbath over conventional tick control methods for *A. hebraeum, R. microplus and R. decoloratus* ticks in Lowveld cattle and for most tick species (*A.hebraeum, R. decoloratus, R. appendiculatus and R. evertsi evertsi*) found in goats in the highveld and in Lowveld sites. One the other hand, in cattle from the Highveld site, footbath did not perform better than conventional methods to control the main tick species found there (i.e. *R. decoloratus*). Similarly, footbath did not perform better than conventional methods to control Hyalomma tick species (i.e. *H. truncatum* and *H. rufides*) found in goats and cattle in the Highveld and Lowveld sites. The study also shows patterns of seasonal variation (i.e. difference between the rainy season and the dry season) in ticks infestation rates that differ depending on site, host species and tick species. These main findings are discussed below.

The study aimed to evaluate the effectiveness of an acaricide footbath in controlling tick infestation on cattle and goats in Zimbabwe. Amitraz was selected because it is the most widely used acaricide in the country and was recommended by DVS. Originally developed in Burkina Faso, this method successfully controlled *Amblyomma variegatum,* the dominant tick species in that region, using deltamethrin, flumetrin and alphacymethrin acaricides (Stachurski and Lancelot, 2006). While these West African studies provide a useful reference, direct comparison is limited by ecological differences between regions, including climate, altitude, vegetation structure, tick species composition, and livestock management systems. Our study extends this approach to Southern Africa under contrasting ecological conditions (Highveld and Lowveld) using a different acaricide formulation, and evaluates its broader efficacy across multiple tick species, thus providing novel data from a region where such trials were previously undocumented. Comparable challenges related to tick infestation dynamics and control have been reported in other developing regions, including Pakistan (Abbas et al., 2025) and across multiple Asian countries (Byun et al., 2025), highlighting the importance of evaluating context-specific and resource-efficient tick control strategies.

Baseline tick counts in cattle in the Lowveld were high (data shown in Table 1 and Fig. 2) because farmers had missed two dipping sessions due to challenges with refilling dip tanks, making the acaricide footbath intervention both timely and justified during a water shortage. In most communal areas in Zimbabwe, water availability in the dry season is particularly challenging, and water resources are prioritized for humans rather than livestock (Akinmoladun et al., 2019). In marginalised regions, the limited water surplus which is insufficient for replenishing dip tanks can be effectively utilized for footbaths which require significantly less water (50 times less). This water efficiency addresses a key constraint identified in national dipping programs and offers a realistic, community-adaptable solution.

During the present study, *A. hebraeum* was consistently identified at Gonakudzingwa and Matopos but not at Marirangwe while *A. variegatum* was not found on any site. Currently, *A. hebraeum* is the predominant bont tick species which is found in almost all parts of the country though it still has higher densities in the south-eastern Lowveld (Shekede et al., 2021). Although *A. variegatum* is mainly distributed in the north western regions of the country, its range extends eastward onto the highveld, but does not reach the eastern highlands (Sungirai et al., 2017; Shekede et al., 2021). The south eastern Lowveld of Zimbabwe constitutes a historically endemic region for heartwater, with *A. hebraeum* serving as the primary vector (Peter et al., 1999; Caron et al., 2013). The increase infestation by *A. hebraeum* observed in goats in dry season was mainly due to the nymphs which are active during winter and prefer goat/small ruminant hosts. Although *A. hebraeum* infestations persisted despite treatment, the method demonstrated superior efficacy compared to the conventional plunge dipping technique, highlighting its potential as an alternative or complimentary tick control strategy.

Beyond bont ticks, this study also assessed for the first time the impact of footbaths on coexisting tick species transmitting major TBDs such as theileriosis, babesiosis, and anaplasmosis. Seven additional tick species were identified in the Lowveld, five of which showed significant reduction in infestation under the footbath treatment (Table 3). Among these species *R. microplus* is a highly invasive species with documented history of acaricide resistance (Rodriguez-Vivas et al., 2018) that transmits the more virulent cerebral form of babesiosis caused by *Babesia bovis* (Madder et al., 2011; Rodriguez-Vivas et al., 2018; Jain et al., 2020a, Jain et al., 2020b) and has displaced *R. decoloratus* in other parts of the region (Tønnesen et al., 2004; Horak et al., 2009; Dzemo et al., 2023). This study has evidence of sympatry of these species and both were successfully controlled by the footbath in cattle. Reduced *R. microplus* infestation under the footbath treatment was somewhat unexpected since *Rhipicephalus* are questing ticks that are not expected to attach on their hosts's feet. This may be attributed to chemical diffusion occurring when cattle fold their treated limbs when resting, bringing them into contact with the lower abdomen—a primary predilection site for adult *Rhipicephalus* (*Boophilus*) ticks (Horak et al., 2018). Alternatively, it may reflect on changes in tick attachment routes during the dry season, when reduced grass cover forces questing ticks to reduce questing height and thus attach via the feet rather than vegetation tips, thereby increasing their exposure to the acaricide footbath*.*

However, despite the observed reduction in tick infestation, the continued presence of adult *Rhipicephalus (Boophilus)* ticks in weekly collections indicates a potential level of resistance to amitraz in the Lowveld, similar to what has been suggested in the Highveld. Since *R. microplus* and *R. decoloratus* are one-host ticks, their adults emerge on the same host approximately 2–3 weeks after the attachment of the larvae, resulting in prolonged exposure to acaricides, and thereby increasing selection pressure for resistance. Whereas with foot-bathing, these ticks could have been missed when they attached through questing on higher vegetation and attached via other animal body parts than the feet, the plunge dipping should have killed the larval and nymph stages if the tick populations were sensitive to amitraz. Nevertheless, in the absence of formal resistance testing, any inference of potential amitraz resistance remains speculative and requires confirmation through standardized laboratory bioassays.

The acaricide footbath was postulated to be effective in controlling *Hyalomma* ticks due to their active host-seeking (hunting) behaviour and their tendency to attach at predilection sites such as the interdigital spaces and fetlocks of small ruminants, which are directly targeted by the footbath treatment (Kok and Fourie, 1995; Nyangiwe and Horak, 2007; Hove et al., 2008). The acaricide footbath technique has also been reported in South Africa as a method for controlling tick-associated lameness in wool sheep and Angora goats (Spickett and Fivaz, 1992), although limited detail was provided. The present study did not find any strong evidence that footbath could be more effective than conventional dipping in controlling cattle or goat infestation by *H. truncatum* or *H. rufipes*. However, *Hyalomma* ticks were recorded in low numbers so that it is impossible to draw any robust conclusion.

Animals treated via acaricide footbath were occasionally housed with untreated animals. The detachment and transfer of ticks from the feet to nearby animals has previously been documented (Stachurski, 2000) and could thus result in limited cross-infestation that would homogenize infestation rates among animals irrespectively of their treatment group. Tick burdens remained however significantly lower on foot-bathed animals for not only *Amblyomma* ticks but other tick species too, indicating that the footbath treatment was effective.

The Highveld study site of Marirangwe lies within the small-scale commercial sector, where reliable and consistent tick control programs are in place. This resulted in lower tick infestation levels than in the communal land area of Gonakudzingwa, except for *R. decoloratus.* In Marirangwe, farmers implemented the 5–5-4 tick control method, designed to manage *R. appendiculatus* during theileriosis outbreaks. This resulted in reduced *R. appendiculatus* tick counts during the rainy season, and only sporadic tick detections during the dry season, when the spraying frequency was decreased to once per week. However, the observed *R. appendiculatus* infestation in this study may not truly reflect the footbath's effectiveness on this tick in cattle, as tick grease was also specifically applied to the ears to limit infestations and reduce the risk of theileriosis outbreaks increasingly affecting farmers.

The observation of only a few *A. hebraeum* at Marirangwe may suggest that the species may not yet be established in the area, aligning with previous findings (Bournez et al., 2015; Makuvadze et al., 2020). It is also likely that the intensive dipping programs currently in place to control theileriosis outbreaks have effectively suppressed *A. hebraeum* populations. To improve the robustness of the findings, the study should be replicated in a fully communal area setup, with multi-tick species infestations.

The trial followed DVS recommendations, using emulsified acaricide concentrations of 50% in the Lowveld and 25% in the Highveld. The amitik (Amitraz) chemical used in this study has a proven residual effect lasting up to 7 days, depending on environmental conditions (Ahmed and Mume, 2023). Foot-bathing was conducted every 2–3 days, thus within the 7-day window. The inability to control the high numbers of *R. decoloratus* ticks at Marirangwe suggests potential resistance to amitraz, likely driven by its continuous intensive use under the 5–5-4 application regimen and the lower concentration of 25%, compared to the 50% used at Gonakudzingwa. Low-level amitraz resistance of *Rhipicephalus* subgenus *Boophilus* and *R. appendiculatus* ticks has previously been demonstrated in Zimbabwe using the larval packet test (Chitombo et al., 2021; Makuvadze et al., 2020; Nemaungwe, 2023) and molecular typing (Sungirai et al., 2018). Preliminary studies by DVS suggested a negligible resistance level for amitraz in a few areas around the country (Nemaungwe, 2023). The disparity in footbath effectiveness against certain *Rhipicephalus* species between Lowveld and Highveld cattle could be partly explained by the variation in amitraz concentrations applied. The difference could also imply that the footbath need to be coupled with a strongly effective acaricide to achieve its maximum control potential evidenced by the better results realised in prior studies (De Meneghi et al., 2016; Stachurski and Lancelot, 2006).

Seasonal variation in tick infestation should also be considered in the light of seasonal shifts in cattle grazing habitats. During the dry season, cattle grazed in and around arable lands post-harvesting, whereas in the rainy season they grazed in bushes and thickets. These differing habitats may harbour different tick species. For instance, *Amblyomma* species are known to prefer tree-shaded grounds (Norval et al., 1992). Moreover, the rainy season allows the growth of tall grasses around grazing areas, providing a suitable habitat for questing ticks like *R. appendiculatus*, *R. decoloratus, and R. microplus*. During the dry season, however, the absence of tall grasses in the veld forces even questing tick species to rely on low height ground debris or moribund vegetation for questing (Estrada-Peña et al., 2013; Leal et al., 2020).This behaviour increases the likelihood of these ticks attaching to animal hosts via the feet, similar to the attachment strategy of hunting tick species. These findings suggest that the acaricide footbath is best suited for use during the dry season in these semi-arid areas, when grass cover is minimal and water availability is limited.

The study also demonstrated that goats had persistently lower tick numbers than cattle, suggestive of their lower attractiveness to the tick species present in the study area but that they could host *Rhipicephalus microplus* ticks, which are ordinarily considered cattle-specific (Horak et al., 2018).

Although tick populations are generally expected to peak during the rainy season, this study found higher numbers of *R. decoloratus* and *R. microplus* in cattle during the dry season, as well as increased *A. hebraeum* infestations in goats, contrary to previous reports that documented peak populations during the rainy season (Manunure et al. 2021; Mooring et al., 1994; Norval et al., 1991). This could be due to relaxed mandatory plunge dipping in the dry season. During this period, dipping frequency is reduced to fortnightly nationwide, in contrast to the weekly treatments applied in the rainy season. The detection of *Amblyomma hebraeum* nymphs on goats in noteworthy, as they are highly competent vectors of *Ehrlichia ruminantuim*, the causative agent of heartwater. Nymphal abundance during winter agrees with what was reported by Jongejan et al. (2020) and Hove et al. (2008). Their control should be considered a priority in this periods (Bryson et al., 2002; Nyangiwe and Matthee, 2025).

### 4.1 Limitations

There were several limitations to this study. Firstly, the choice of acaricide and its dilution for field application was determined by the DVS, which regulates national usage (DVS, 2015). Consequently, different concentrations of amitraz were used in the two ecological zones: 50% in the lowveld and 25% in the highveld. There were no prior studies assessing amitraz resistance levels in the specific study areas and reliance was mainly on guidance from previous studies conducted in different communal land settings within the country (Makuvadze et al., 2020; Chitombo et al., 2021; Nemaungwe, 2023). In addition, other acaricide groups that might have yielded greater efficacy in a footbath system were not permitted for use in this study.

Secondly, the lack of separate overnight pens and the inability to isolate treated and control groups during transit after footbath may have introduced bias due to cross-infestation by ticks. Shared housing could have facilitated limited tick transfer between treatment and control animals, potentially homogenizing infestation levels and thereby underestimating the true effect of the footbath treatment. Despite this conservative bias, significant reductions in tick counts were consistently observed on foot-bathed animals, supporting the robustness of the observed treatment effects.

Thirdly, the three study sites had different vegetation types, harbouring different tick species and habitat conditions. Consequently, tick behaviour may have been influenced by varying abiotic factors.

## 5 Conclusion

The study revealed that amitraz acaricide footbaths are effective in controlling *A. hebraeum* on both cattle and goats, and can also reduce infestations of other common tick species, including *R. decoloratus* and *R. microplus*, particularly in lowveld areas with limited grass cover. In the highveld, reductions were observed for *R. decoloratus* and *R. appendiculatus* on goats, although efficacy was lower on *R. decoloratus* and *H. truncatum* in cattle, suggesting potential acaricide resistance in some populations. These results highlight the importance of optimizing acaricide concentration and rotation, application frequency, and monitoring for resistance. This study underscores the potential of the acaricide footbath to enhance tick management programs, especially in regions facing water shortages or where alternative methods are less effective. Based on the findings, footbathing provides a more convenient and less stressful application method but should be regarded as a strategic supplementary control measure, rather than a complete replacement for plunge dipping, particularly in areas with high infestations of multi-host tick species that preferentially attach to the upper body. We therefore recommend routine use of footbaths during the dry season in communal livestock systems, integration with existing dipping or spraying schedules, and the use of footbaths as a stand-alone control method for small ruminants, where predominant tick species mainly infest the lower limbs, which constitute their primary predilection sites. Molecular confirmation of tick species and acaricide resistance was not performed and should be addressed in future studies to ensure long-term effectiveness.

## CRediT authorship contribution statement

**Obey Daga:** Writing – review & editing, Writing – original draft, Visualization, Validation. **Thokozani Hove:** Writing – review & editing, Supervision, Methodology, Formal analysis. **Silvester Chikerema:** Supervision, Methodology, Conceptualization. **Vladimir Grosbois:** Supervision, Project administration, Methodology, Formal analysis, Data curation, Conceptualization. **Christopher Gadzirai:** Methodology, Investigation, Conceptualization. **Frédéric Stachurski:** Writing – original draft, Methodology, Conceptualization. **Mathieu Bourgarel:** Project administration, Funding acquisition, Conceptualization. **Laure Guerrini:** Writing – review & editing, Writing – original draft, Supervision, Resources, Project administration, Methodology, Funding acquisition, Data curation, Conceptualization.

## Declaration of competing interest

The authors declare there is no conflict of interest.
